# Germs and germlines: how “public” B‐cell clones evolve in the gut

**DOI:** 10.1111/imcb.12340

**Published:** 2020-05-16

**Authors:** Kylie R James, Hamish W King

**Affiliations:** ^1^ Wellcome Sanger Institute Wellcome Genome Campus Hinxton CB10 1SA UK; ^2^ Centre for Immunobiology Blizard Institute Queen Mary University of London London E1 2AT UK

## Abstract

Chen *et al.* describe how B‐cell clones observed in the gut of many different individuals (recurrent or “public” clonotypes) are shaped by the combined influences of common microbial antigens and underlying genomic recombination biases.

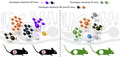

Within gut‐associated lymphoid tissue such as Peyer’s patches, B cells generate high‐affinity antibodies through somatic hypermutation of their immunoglobulin genes in germinal center (GC) reactions. Uniquely, compared with other lymphoid tissues, these GC reactions typically arise in a homeostatic manner rather than as part of an acute immune response. Fittingly, there appears to exist an intimate relationship between the gut microbiota, the maintenance of Peyer’s patch GCs and the subsequent antibody repertoire that they generate, although many open questions remain about B‐cell responses in the gut.

Recently, Chen *et al.* used a high‐throughput genomic sequencing approach to examine the diversity of the immunoglobulin variable (V), diversity (D) and joining (J) genes expressed by B‐cell clones in mouse gut tissue.^1^ Despite the enormous potential combinatorial diversity of antibody repertoires, they found an over‐representation of specific B‐cell clonotypes undergoing maturation in GCs compared with naïve B‐cell populations. Intriguingly, several of these clonotypes were consistently observed across multiple mice, reminiscent of public clonotypes described elsewhere in circulating and gut human B‐cell repertoires.^2^, ^3^ The existence of such recurrent clonotypes engaging in GC maturation indicated strong selection pressures were at play, and Chen *et al.* provide compelling evidence that both antigen‐dependent and antigen‐independent mechanisms shape the existence of public clonotypes.

The requirement of antigen for chronic Peyer’s patch GCs has previously been demonstrated by the restoration of Peyer’s patch GC reactions in germ‐free mice after vaccination^4^ or colonization with commensals.^5^ Chen *et al.* build on these and other studies by comparing the antibody repertoires of normal laboratory (specific pathogen‐free) mice and germ‐free mice colonized with specific pathogen‐free mouse microbiota (Figure 1). While germ‐free mice appear to lack some of the key public clonotypes identified in the control mouse population, the introduction of the specific pathogen‐free mouse microbiota into germ‐free mice resulted in an expansion of recurrent B‐cell clonotypes, strongly implicating microbial‐derived antigens in the expansion of these public clonotypes.^1^ Importantly, the authors sequenced matched heavy‐ and light‐chain antibody sequences of some recurrent clonotypes using single‐cell RNA‐seq and validated that two such clonotypes shared between the germ‐free and control mice were able to bind specific microbial glycans and that this affinity was acquired through somatic hypermutation. These observations not only intimately link the selection and affinity maturation of homeostatic B‐cell responses in the gut with the microbiome but also demonstrate that at least some public clonotypes found between individuals in homeostatic conditions are driven by exposure to common microbial antigens.

**Figure 1:**
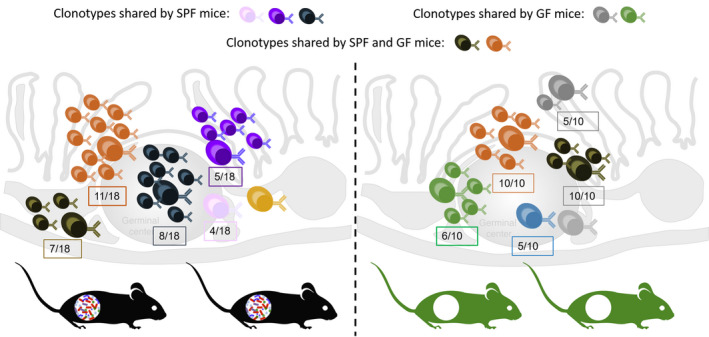
B‐cell clonal repertoires of the gut are selected by microbiome and genomic recombination biases. Schematic depicting key experiments by Chen *et al.* to show enrichment of clonotypes in germinal center (GC) Peyer’s patches (PPs) of specific pathogen‐free (SPF; black) mice and germ‐free (GF; green) mice. Enrichment of dominant clonotypes is shown as a fraction of mice containing that clone.

However, and perhaps most intriguingly, Chen *et al.* propose that the existence of recurrent B‐cell clonotypes is also influenced by underlying genetic biases in the generation of the B‐cell antibody repertoire independent of affinity maturation in the GC. Recurrent clonotypes were identified in both germ‐free mice and mice lacking the ability to undergo affinity maturation (Activation‐induced cytidine deaminase deficient), showing that public clonotypes in the gut, and perhaps more broadly, can arise independently of antigen‐driven affinity maturation. This is reminiscent of another recent study that found evidence for public clonotypes in human cord blood B cells, prior to any affinity maturation.^3^ Furthermore, many recurrent clonotypes in mouse gut tissue not only share V and J immunoglobulin gene usage across multiple mice, but also exhibit canonical sequences in their complementarity‐determining region 3,^1^ which typically confers antigen affinity. Computational modeling revealed that the complementarity‐determining region 3 sequences of recurrent B‐cell clones had higher inherent generation probabilities for their junctional diversity than expected by chance. Given that the complementarity‐determining region 3 sequence is the recombination product of immunoglobulin V(D)J genes present in an individual’s germline and the addition of nontemplated nucleotides between the DNA of V(D)J genes, the authors propose that inherent biases during V(D)J recombination influence the nature of public clonotypes. It is tempting to speculate that such increased likelihoods of generating or selecting for specific clonotypes with higher baseline affinities toward common microbial antigens may have evolved through previously described genetic mechanisms such as distancing between V, D and J genes; *cis*‐regulatory element function; copy number variation and genetic variation in the recombination signal sequences that guide V(D)J recombination at the immunoglobulin loci.^6^, ^7^, ^8^

The prevalence of commonly occurring B‐cell clonotypes in the homeostatic mouse gut therefore appears to be shaped by the combined influence of underlying genetic biases and exposure history to common microbial antigens, rather than resulting solely from stochastic processes. While it is unclear how these findings from inbred laboratory mice translate to the more genetically and microbially diverse human population, this study raises important questions about human B‐cell clonal dynamics in both the healthy and diseased gut. In general, B‐cell clonal dynamics throughout the human gut remain poorly understood,^9^ and despite recent efforts from us^10^ and others, the public B‐cell clonal repertoire of the human gut has yet to be examined in sufficient detail to draw many direct parallels. As more information on human gut B‐cell repertoires is acquired, it will be interesting to examine whether the presence or absence of public B‐cell clonotypes are associated with genetic variation at the immunoglobulin loci, immunodeficiency‐ or autoimmunity‐associated disease and colonization by specific commensal or pathogenic microbiota in the gut. By building a better understanding of how the nature and efficacy of local B‐cell responses in the gut are shaped by the complex interplay between an individual’s microbiota and underlying genetic variation we may gain new insights into the pathophysiology of common diseases in the gastrointestinal tract such as inflammatory bowel disease.
